# A feasibility randomised controlled trial of a Fibromyalgia Self-management Programme for adults in a community setting with a nested qualitative study (FALCON)

**DOI:** 10.1186/s12891-022-05529-w

**Published:** 2022-07-11

**Authors:** Jennifer Pearson, Jessica Coggins, Sandi Derham, Julie Russell, Nicola E. Walsh, Erik Lenguerrand, Shea Palmer, Fiona Cramp

**Affiliations:** 1 Faculty of Health and Applied Sciences, University of the West of England, Glenside Campus, Bristol, UK; 2 Brownsword Therapies Centre, Royal United Hospitals Bath NHS Foundation Trust, Bath, UK; 3 Musculoskeletal Research Unit, Translational Health Sciences, Bristol Medical School, University of Bristol, Bristol, UK; 4 Centre for Care Excellence, Coventry University and University Hospitals Coventry & Warwickshire NHS Trust, Coventry, UK

**Keywords:** Fibromyalgia, Feasibility randomised controlled trial, Self-management, Community

## Abstract

**Background:**

Fibromyalgia is a condition associated with widespread musculoskeletal pain, fatigue and sleep problems. Fibromyalgia treatment guidelines recommend non-pharmacological interventions and the development of self-management skills. An example of a programme that fits these guidelines is the Fibromyalgia Self-management Programme (FSMP) which consists of one 2.5-hour weekly session over six successive weeks and includes education about fibromyalgia, goal setting, pacing, sleep hygiene and nutritional advice. The FSMP is currently provided in a secondary care hospital setting and co-delivered by a multidisciplinary team. Delivery in a primary care setting has the potential to improve the accessibility of the programme to people with fibromyalgia. Therefore, this feasibility study aimed to determine the practicality and acceptability of conducting a future definitive randomised controlled trial of the FSMP in a community setting.

**Method:**

An exploratory, parallel-arm, one-to-one, randomised controlled trial. Participants were recruited from general practices across South West England, and the FSMP was co-delivered by physiotherapists and occupational therapists across two community sites. To determine the outcome measures for a future definitive trial several were tested. The Revised Fibromyalgia Impact Questionnaire, Arthritis Self-Efficacy Scale-8, Chalder Fatigue Scale, Short form 36, 5-Level EQ-5D version and Jenkins Sleep Scale were collected at baseline, 6 weeks and 6 months. Semi-structured interviews were conducted with patient participants, occupational therapists and physiotherapists to explore the acceptability and feasibility of delivering the FSMP in a community setting.

**Results:**

A total of 74 participants were randomised to the FSMP intervention (*n* = 38) or control arm (*n* = 36). Attrition from the trial was 42% (31/74) at 6 months. A large proportion of those randomised to the intervention arm (34%, 13/38) failed to attend any sessions with six of the 13 withdrawing before the intervention commenced. The proportion of missing values was small for each of the outcome measures. Three overarching themes were derived from the interview data; (1) barriers and facilitators to attending the FSMP; (2) FSMP content, delivery and supporting documentation; and (3) trial processes.

**Conclusion:**

It is feasible to recruit people with fibromyalgia from Primary Care to participate in a randomised controlled trial testing the FSMP in a community setting. However, improvement in trial attrition and engagement with the intervention is needed.

**Trial registration:**

The trial is registered with ISRCTN registry and was assigned on 29/04/2019. The registration number is ISRCTN10824225.

## Background

Fibromyalgia (FM) is a common condition that affects over 5% of the UK population [1, 2] and has a higher incidence in females than males [3]. FM symptoms include chronic widespread pain, fatigue, sleep problems, stiffness, cognitive dysfunction and psychological distress [4, 5]. The condition is often linked to high levels of physical disability, increased use of healthcare resources and lost workdays [6–8]. The guidelines for the treatment of FM recommend that treatment should focused on non-pharmacological interventions rather than pharmacogological interventions [9–11]. The best evidenced non-pharmacological interventions include; aerobic exercise, hydrotherapy, relaxation, cognitive behaviour therapy (CBT) and patient education [9–11]. However, it is also important that individuals with FM develop the knowledge and skills needed to independently self-manage their condition. There is a convincing argument in using self-management interventions for treatment of long-term conditions, such as FM, as they have been shown to improve participant engagement, physical symptoms and function, self-efficacy and mood and reduce health related costs [12–14]. Other research investigating FM self-management within a community setting found an improvement in confidence to manage symptoms of pain, short-term reduction of FM symptoms, decreased fatigue, and a drop in the number of General Practitioner FM visits [15].

Members of the Rheumatology Therapies team at the Royal United Hospitals Bath (RUHB) developed the Fibromyalgia Self-Management Programme (FSMP). The programme is a non-pharmacological, multidisciplinary exercise and education group intervention that aims to provide condition-specific, patient-centred education and exercise advice and support the development of self-management skills. The FSMP is delivered in 2.5 hour sessions over six successive weeks and includes sessions on education about FM, goal setting, pacing, sleep hygiene and nutritional advice. Using Michie Behaviour Change Taxonomy [16], a recent study [17] mapped the FSMP to 22 behaviour change techniques which covered 12 of the 16 main behaviour change domains. The study found key factors that facilitated behaviour change included FM education alongside patient-focused goal setting. The findings also suggest that delivering the programme in a group setting was perceived as beneficial as individuals shared, with others, their experiences of diagnosis and the management of their symptoms [17]. The findings show the Capability, Opportunity, Motivation and Behaviour (COM-B) model [18] is a useful theroectical framework to understand how the FSMP intervention works in practice.

Until recently, the FSMP has been provided in a secondary care hospital setting and co-delivered by a multidisciplinary team. Local audit data reports good patient satisfaction and improvements in self-efficacy to manage FM symptoms. However, this has yet to be formally evaluated using robust quantitative research methods. The UK government plans to increase investment in community-based healthcare services to deliver care for those living with long-term conditions closer to home. Therefore, some services that were once provided in acute hospitals will now be transferred to the community [19, 20]. A recent review reported no clear evidence of the benefit of treating FM in secondary care. The authors recommend developing a new model of care for FM, and highlight the potential benefits of providing care in a primary care setting [21]. Transferring the FSMP to a community setting presents opportunities to offer specialised care closer to home and determine the clinical and cost-effectiveness.

### Aim and objectives

This feasibility study aimed to determine the practicality and acceptability of conducting a future definitive randomised controlled trial (RCT) of the clinical and cost-effectiveness of a community-based FSMP.

The specific objectives were to:

- Determine the feasibility of training Band 6 PTs and OTs to deliver the FSMP in the community;
- Explore the feasibility of recruiting adults with FM to the trial from primary care;
- Assess the feasibility of collecting a range of outcome data to identify the primary outcome for a future trial;
- Assess the feasibility and acceptability of collecting health economic data;
- Determine the recruitment rate and calculate the sample size for a full trial
- Determine the safety of delivering the FSMP in a community setting
- Understand the patient and health professional acceptability of delivering the FSMP in the community.

## Methodology

This feasibility randomised controlled trial followed a pre-specified protocol [22] (ISRCTN registration 10,824,225 was assigned on 29/04/2019). An exploratory, parallel-arm, one-to-one, RCT design was used. The feasibility trial was conducted over two sites in South West England (SW England) between 17th July 2019 and 11th December 2019. Ethical approval was obtained by Yorkshire & the Humber - South Yorkshire Research Ethics Committee (18/YH02/63) on 9th August 2018. This study adhered to the principles defined in the declaration of Helsinki [23]. The Consolidated Standards of Reporting Trials (CONSORT) checklist for randomised pilot and feasibility studies was used to provide a complete and comprehensive report of this study [24].

### Patient and public involvement (PPI)

Two patient Research Partners (RP) were recruited and contributed to the study conception, design and interpretation of findings. The PPI perspective was represented at all trial management meetings, and both RPs gave invaluable guidance and support to the research team throughout the development and delivery of the study.

### Participants and the recruitment process

Participants were recruited from research-active general practices (GP) across SW England. The practice manager, research nurse or general practitioner at consenting GP sites, using GP read codes, conducted a database search for patients diagnosed with FM and aged 18 and over. From the identified potential trial participants, a member of the GP team screened for eligibility and suitability (for example, excluded if recently bereaved or under medical investigation for serious pathology). The GP then sent an information pack by mail, which included; an invitation letter from the GP; a detailed participant information leaflet (PIL); the contact details of the research team; a reply slip, and a prepaid envelope. Interested participants returned the reply slip or contacted the research team by telephone or e-mail.

Potential trial participants were then screened over the telephone by the Chief Investigator (CI) (JP) for further eligibility criteria and were excluded if they had a General Anxiety Disorder Assessment (GAD-7) score > 15 [25], had previously attended the RUHB FSMP or another pain management programme, required a carer to enable attendance at the FSMP or an interpreter to communicate in English (Table 1). The eligibility criteria used reflect the criteria followed at the RUHB. Once eligibility was established, the CI discussed the PIL with the potential participant providing information about the study, the process of randomisation, their involvement and trial participation. Initial verbal consent was then obtained over the telephone. Full written consent was also obtained from participants prior to submission of any data.

**Table 1:** Inclusion and exclusion criteria

Inclusion criteria	Exclusion criteria
Adults aged ≥18 years	Rheumatoid arthritis diagnosis
Fibromyalgia diagnosis	GAD-7 score > 15
Willing to take part in a group-based intervention	Previously attended the RUHB FSMP or another pain management programme
Ability to travel to attend the group sessions	Requires a carer to attend
	Requires an interpreter to communicate in English

### Intervention – FSMP

The intervention consisted of a 6 week, FM condition-specific group programme delivered by a PT and an OT. The intervention was delivered four times at two selected community sites in SW England (two courses per Trial site). Trial site A was situated in a community GP in a city, and Trial site B was located in a rural community hospital. The FSMP comprised a 2.5 hour weekly group session over six consecutive weeks. Each week, the course focused on supporting individual self-management skills by increasing knowledge and understanding of the condition, medication, goal setting, pacing, dietary advice, sleep hygiene, relaxation, and exercise. All participants attended the exercise sessions but could opt-out at any time if they needed a rest. Participants also received a booklet that contained information on the programme, information about FM and self-management strategies, online links to other relevant resources (for example, Versus Arthritis (https://www.versusarthritis.org/about-arthritis/conditions/fibromyalgia [26]) and Fibromyalgia action UK (https://www.fmauk.org [27])) and worksheets. The participants were encouraged to take notes, complete the worksheets and to keep the booklet on completion of the programme. Participants in the intervention arm continued to receive usual care from their GP throughout the trial.

### Therapist training

The FALCON training programme was developed by the research team and delivered by RUHB clinical lead occupational therapist (SD) and physiotherapist (JR), both of whom were instrumental in creating the existing secondary-care FSMP at the RUHB. Four physiotherapists and two occupational therapists attended a two-day training programme at the RUHB in February and March 2019. Two additional therapists were trained to ensure that the intervention could be delivered if one or more therapists were absent. The training consisted of an overview of the study and the FSMP, including documentation and administration, strategies when managing groups, and all content included in the FSMP (diagnosis, acceptance and the grief cycle, activity balance and goal setting, pain, deconditioning and re-conditioning, mindful movement, mood, sleep, relaxation, communication skills, medication and healthy eating). To offer further support and to ensure intervention fidelity JD or SD attended two of the six FSMP community sessions. If the delivering therapists had any concerns, they could contact either facilitator by telephone or e-mail.

### Usual care

Participants continued to receive usual care from their GP, they were not discouraged from seeking additional healthcare for their FM symptoms but were advised to contact the research team if they were referred to a pain self-management programme. Once data collection was complete, participants randomised to the control arm were sent an FM information leaflet designed by the research team. The leaflet provided information on FM, current treatments and information about local support groups.

### Outcomes

The outcomes that were of particular relevance for the feasibility of a future trial included the number of patients identified with FM in primary care, percentage of FM patients deemed eligible, recruitment to the feasibility trial as a percentage of those contacted, number of analysable completed patient-reported questionnaires, attendance at the FSMP and number of patients who drop out of the FSMP.

To determine the outcome measures for a future definitive trial, several outcomes were tested. All clinical outcome measures were patient-reported. Participants randomised to the intervention arm returned the outcome measures by post at baseline and 6 months but completed the 6 week outcome measures on-site at the end of the intervention. Those who did not attend were sent the questionnaires by post. Participants in the control arm were sent the outcome measures by post for all three time-points.

To identify a suitable and feasible primary outcome measure for the definitive trial, the research team collected a range of FM symptom-based, quality of life (QoL) and self-management specific outcome measures. To assess the impact of FM symptoms, the Revised Fibromyalgia Impact Questionnaire (FIQR), a validated outcome measure to evaluate FM, was used [28]. As fatigue is a significant symptom that people affected by FM find burdensom [29, 30] the Chalder Fatigue Scale (CFS) Questionnaire was used [31], which has previously been used in the FM population [32]. To monitor changes to QoL, the SF-36 was used and has been validated for use in Primary Care [33]. The EQ-5D-5L was also used to measure QoL [34]. Self-efficacy data were also collected as it can predict changes in self-management related health behaviours and increased levels of self-efficacy are closely linked with effective self-management of FM [35–37]; The Arthritis Self-Efficacy Scale-8 (ASES-8) was used as it is a reliable and valid measure for FM [38, 39]. To explore potential changes in sleep the Jenkins Sleep Scale (JSS) was included [40, 41].

To assess the feasibility of collecting health economic data, the Client Service Receipt Inventory (CSRI) was adapted for FM to collect health and social care use [42, 43]. It was proposed that Health economic data was to be collected by the RA (JC) attending each participating GP practice and conducting an electronic medical record review of consultations, prescriptions and onward referrals to other services over the previous 6 months. However, due to restrictions during the COVID-19 pandemic data were collected using an alternative method. An appropriate member of staff at the participating GP practices were sent an electronic medical records review form which they completed and returned securely to the research team. An ethical amendment was obtained from Yorkshire & the Humber - South Yorkshire Research Ethics Committee (18/YH02/63) on the 7th of July 2020 to allow the self-reported non-identifiable questionnaires to be stored securely at the RAs (JCs) home during the COVID-19 pandemic.

### Sample size

To account for loss-to-follow, missing data and estimate parameters such as the participation or completion rates, and those required to derive the sample size for the main trial with enough precision. The trial aimed to recruit a total sample size of 70 participants, with a minimum of 35 in each arm.

### Randomisation and allocation concealment

Participants were randomised to either the control arm or the FSMP using a parallel 1:1 study design. Randomisation was stratified by Trial site and cohort. Randomisation was conducted independently by the Research and Development (R&D) team at the RUHB using online software [44]. To preserve concealment, a list of non-identifiable participant ID numbers was sent to the R&D team who subsequently produced a randomised sequenced list. The R&D team did not have any contact with the participants and did not have access to confidential or clinical data. Following randomisation, the research team were informed of the participant allocation. The participants were informed by the research associate (JC) once their baseline data had been received.

### Blinding

Participants in the intervention arm attended the FSMP, therefore participant blinding was not possible. The therapists delivering the FSMP, the research associate (JC) and the data analyst (SP) were also unblinded to participant allocation.

### Nested qualitative study

A nested qualitative study was conducted to understand the acceptability of FSMP and whether it was feasible to be delivered in a community setting. After completing the programme, all patient participants randomised to the intervention and the therapists delivering the programme were invited to take part in semi-structured interviews to share their experiences of the FSMP. Of those patients who responded, participants were purposively selected based upon key characteristics including Trial site, age, gender, the severity of FM and attendance at the FSMP. The semi-structured interviews were conducted by JC (a female Sports Scientist with qualitative methodological training).

### Quantitative analysis

Quantitative descriptive analysis included the number and percentages of participants approached, recruited and retained in the study and the completion of the intervention and outcome data. The final data included reasons for non-participation, withdrawal, missing data and noncompliance with the protocol with the emphasis on how these may impact on the full-scale trial. These rates are presented by trial arms to investigate any differences requiring particular attention in the design of the main trial. Deviations from the protocol were recorded and reported by relevant categories to identify areas requiring particular attention during the design of the main trial. Descriptive statistics, including means and standard deviations, were used to analyse the patient-reported outcome measures. Sample size calculations for a full RCT were performed using G*Power (version 3.1.9.4), 90% power, and a two-sided alpha of 5% [45]. The data completeness of the different outcomes to identify those with the highest completion rate and candidate measures for the main trial were also reported. A similar descriptive statistics approach was used for the health economic data.

### Qualitative data analysis

The qualitative research was underpinned by a qualitative description approach [46]. All interviews were audio-recorded and transcribed verbatim. All transcripts were read, checked for accuracy and anonymised to remove identifying features. Each transcript was then given a unique ID and pseudonym and uploaded to NVIVO software [47]. JC then coded the transcripts with a selection (*n* = 3) doubled coded (JP) to ensure all data was captured and the interpretation was refined. The codes were then grouped into categories and thematically analysed [48, 49] to develop a comprehensive understanding of the acceptability of the intervention in the community, feasibility of the RCT and identification of important clinical outcomes.

Six of the FSMP sessions were audio-recorded for fidelity purposes. A coding framework was developed by the research team and used as a fidelity assessment tool [50, 51]. The tool included key areas of the training course and the therapist’s and patient’s manuals, and sections of the raw audio files were coded to the framework. Data was reviewed at the end of the study (by JC), exploring whether the therapists delivered the course in congruence with delivery of the intervention in secondary care.

### Data management

All data were managed in accordance with the 2018 Data Protection Act [52]. All serious adverse events (SAEs) and adverse events (AEs) were reported to the CI and sponsor and robustly investigated to determine causality [53].

## Results

### Recruitment

Between April and August 2019, 20 General Practices across two sites in SW England invited 1414 patients with an FM diagnosis and aged 18 or over to participate in the study. A total of 217 patients (15%) responded, 198 (14%) were screened for eligibility and 77 (5%) consented to take part. Of the 19 participants who were not screened for eligibility, 42% were unable to contact, 5% were no longer interested in participating in the study, and 53% wanted to participate but there were insufficient places available at Trial Site A. Data were not collected for those who were invited but either declined or did not respond. Three participants withdrew prior to randomisation (unable to attend dates provided, unable to travel the distance to attend the programme and no reason provided). The remaining participants (*n* = 74) were randomised to either the intervention (*n* = 38) or the control arm (*n* = 36). Baseline data for six participants from the intervention arm and two from the control arm were not received, leaving *n* = 66 available for quantitative data analysis (*n* = 32 intervention and *n* = 34 control).

### Attrition

**Fig. 1:**
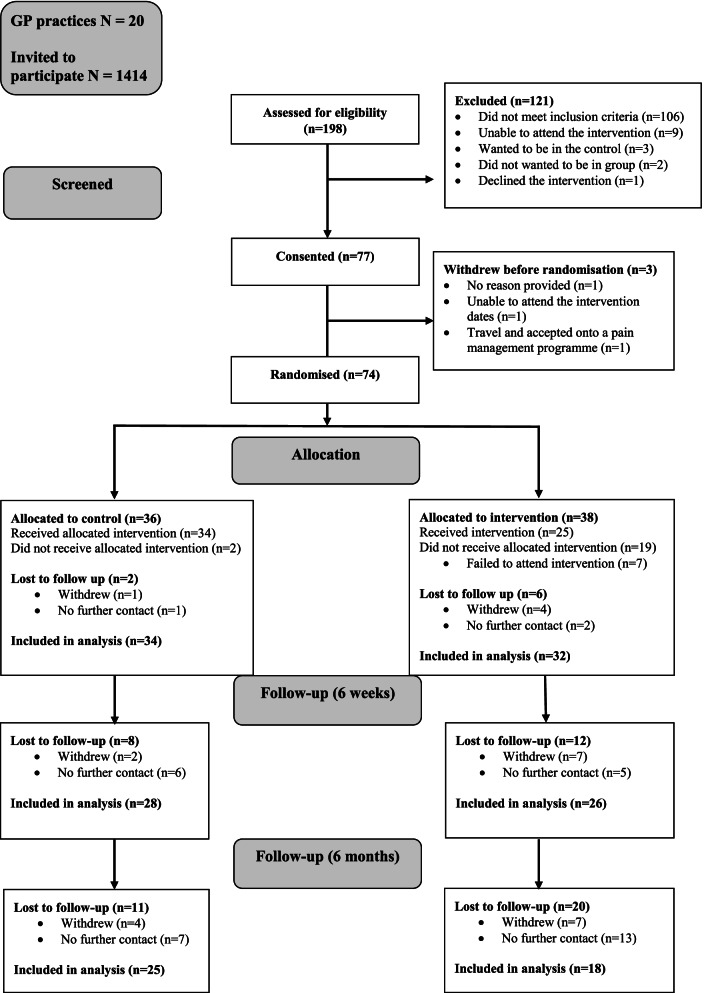
Flow diagram

Overall, total attrition from the trial was 42% (31/74) at 6 months. Attrition was higher in the intervention arm compared to the control arm (32% versus 22% at 6 weeks and 53% versus 31% at 6 months). See Fig. 1.

A large proportion of those randomised to the intervention arm (34%, 13/38) failed to attend any sessions (six of the 13 withdrew before the intervention commenced). This was particularly noticeable in cohort 2 in Trial site B, where 7/9 participants did not attend any sessions (Table 2). Those who did attend the intervention programme (66%, 25/38), attended a median of four sessions and 80% (20/25) attended three or more sessions.

**Table 2:** Distribution of number of sessions attended by those allocated to receive the intervention (total *n* = 38)

Number of sessions attended	Trial site A		Trial site B		Total
	Cohort 1	Cohort 2	Cohort 1	Cohort 2	
**0**	2	2	2	7	13 (34%)
**1**	0	1	1	1	3 (8%)
**2**	1	0	0	1	2 (5%)
**3**	1	0	1	0	2 (5%)
**4**	1	2	1	0	4 (11%)
**5**	2	1	3	0	6 (16%)
**6**	2	6	0	0	8 (21%)

n.b.: The average travel distances were 2.6 miles for cohort 1 in Trial site A, 7.1 miles for cohort 2 in Trial site A, 4.7 miles for cohort 1 in Trial site B, and 12.5 miles for cohort 2 in Trial site B, cohort 2

### Baseline characteristics

The control arm (n = 34, 3 male and 31 female) were on average older than the intervention arm (*n* = 32, 4 male and 28 female) (Table 3). The intervention arm displayed higher SF36 domain values (indicating better health) than the control arm for Physical Function (PF), Role Physical (RP), Role Emotional (RE) and General Health (GH), but lower values for Bodily Pain (BP). The other SF36 subscales (Vitality (VIT), Mental Health (MH) and Social Functioning (SF)) were broadly comparable between study arms. Data were only included if the participant returned the outcomes total (*n* = 66).

**Table 3:** Baseline characteristics (mean, standard deviation)

	Control (*n* = 34)	Intervention (*n* = 32)
Age (years), mean (SD)	53.18 ± 14.88	51.16 ± 14.71
Female (n, %)	31 (91.2)	28 (87.5)
Male (n, %)	3 (8.8)	4 (12.5)
FIQR	61.63 ± 17.45 ^a^	62.46 ± 20.92
ASES-8	3.74 ± 1.57 ^b^	3.83 ± 1.58
CFS	24.43 ± 6.00	22.76 ± 6.32
SF36 PF	30.00 ± 21.46	37.07 ± 27.96
SF36 RP	2.21 ± 7.20	5.47 ± 13.82
SF36 RE	24.51 ± 36.98	31.25 ± 35.86
SF36 VIT	21.96 ± 17.71	22.86 ± 17.64
SF36 MH	52.12 ± 15.21	54.50 ± 17.65
SF36 SF	38.97 ± 20.36	39.84 ± 27.94
SF36 BP	26.91 ± 17.02	22.58 ± 14.90
SF36 GH	23.27 ± 16.80	33.13 ± 16.64
EQ-5D-5L Health	45.82 ± 21.03	45.38 ± 19.81
EQ-5D-5L Index	0.33 ± 0.30	0.36 ± 0.29
JSS	13.50 ± 4.23	13.10 ± 4.07 ^c^

Revised Fibromyalgia Impact Questionnaire (FIQR), Arthritis Self-Efficacy Scale-8 (ASES-8), Chalder Fatigue Scale (CFS), Short Form 36 Physical Function (SF36 PF), Short Form 36 Role Physical (SF36 RP), Short Form 36 Role Emotional (SF36 RE), Short Form 36 General Health (SF36 GH), Short Form 36 Bodily Pain (SF36 BP), Short Form 36 Vitality (SF36 VIT), Short Form 36 Mental Health (SF36 MH), Short Form 36 Social Functioning (SF36 SF), 5-level EQ-5D version (EQ5DL), Index Jenkins Sleep Scale (JSS)

^a^ The number of complete FIQR outcome measures included in the analysis (Control *n* = 32)

^b^ The number of complete ASES-8 outcome measures included in the analysis (Control *n* = 33)

^c^ The number of complete JSS outcome measures included in the analysis JSS (Intervention *n* = 31)

### Outcomes

#### Adverse events

No SAEs occurred during the study but two AEs were reported to the CI. One of the AEs was a participant’s emotional response to session 2 of the intervention in which the group discussed accepting their FM diagnosis. The therapists contacted the study CI for further advice and support, and the participant continued to attend the intervention. The other AE was a health concern during the intervention period. However, this did not occur in the sessions nor was it related to the intervention or participation in the trial.

### Data completeness

The proportion of missing values was very small for each of the outcome measures, with a maximum total of 1.35% for any single outcome measure. Medical record review forms were completed for a total of 36/66 participants (54.5%). CSRI data were available for 100% of participants who returned questionnaires at each time point (Baseline 66/66, 6 weeks 54/54 and 6 months 43/43).

### Sample size calculations

Findings from this feasibility study and published literature were used to inform decisions about how much change in each outcome measure might be considered clinically relevant. These changes were calculated at 6 weeks and 6 months using data from the feasibility study control arm (patients randomised to the standard care arm only) and derived sample size calculations for a full size trial/RCT accordingly. Further details are presented in Table 4.

**Table 4:** Change in control group outcome scores from baseline and the basis for sample size calculations

Outcome	Week 6	Month 6	Sample Size	
	Change Values, mean ± SD (n) 95% CI	Change Values, mean ± SD (n) 95% CI	6 weeks	6 months
FIQR (max 100)	−1.03 ± 11.28 (*n* = 27)	1.86 ± 9.81 (*n* = 23)	*n* = 294 (147 per group)	*n* = 266 (133 per group)
ASES-8-8 (max 10)	0.13 ± 1.26 (*n* = 28) -	−0.30 ± 1.99 (*n* = 25)	*n* = 266 (133 per group)	*n* = 266 (133 per group)
CFS (max 33)	−1.86 ± 6.68 (*n* = 28)	−1.94 ± 6.76 (*n* = 24)	*n* = 440 (220 per group)	*n* = 346 (173 per group)
SF36 RP (max 100)	6.25 ± 24.18 (*n* = 28)	17.00 ± 34.40 (*n* = 25)	*n* = 1054 (527 per group)	*n* = 1870 (935 per group)
SF36 SF (max 100)	−3.57 ± 16.27 (*n* = 28)	2.08 ± 19.39 (*n* = 24)	*n* = 1054 (527 per group)	*n* = 1458 (729 per group)
SF36 BP (max 100)	−3.84 ± 18.49 (*n* = 28) -	−0.70 ± 15.13 (*n* = 25)	*n* = 732 (366 per group)	*n* = 388 (194 per group)

Revised Fibromyalgia Impact Question (FIQR), Arthritis Self-Efficacy Scale-8 (ASES-8), Chalder Fatigue Scale (CFS), Short Form 36 Role Physical (SF36 RP), Short Form 36 Social Functioning (SF36 SF), Short Form 36 Bodily Pain (SF36 BP)

The minimum clinically important difference (MCID) for FIQR is 14% improvement [25]. This MCID was used on the current results to calculate the estimated treatment effect sizes at 6 weeks (0.38) and at 6 months (0.40). There is no guidance to derive the MCID of the ASES-8. Therefore, it was derived from this feasibility study effect size estimates, ie. 0.4 for 6 weeks and 6 months. The smallest MCID for the CFS is 2.3 [54, 55]. The MCID indicating an improvement in individual SF36 domains was established in patients with SLE as 5 points [56]. This was used as the basis for sample size calculations for each of these three SF36 domains. The results were discussed in detail by the trial steering group, including patient representitives. It was agreed that the primary outcome for a future trial should be the condition-specific FIQR, with the ASES-8 as a secondary mechanism-based outcome to capture changes in self-efficacy.

### Qualitative study

All patient participants who were randomised to the intervention arm and were actively involved in the study (*n* = 32) and four therapists delivering the programme were invited to take part in semi-structured interviews. Of the 32 patient participants invited, 19 responded. Thirteen patient participants were selected and consented to an interview. The qualitative interviews were conducted by JC between September 2019 and January 2020. Patient participant interviews (*n* = 3) took place via telephone (*n* = 6) or face-to-face at the participants home (*n* = 4) or in an interview room at the University (n = 3). Therapist interviews (n = 4) took place at the University (*n* = 2) or individuals’ workplace (n = 2). The duration of interviews ranged from 8 minutes (an interview with a participant who did not attend the intervention) to 127 minutes (mean: 58 minutes). Tables 5 and 6 presents the details of the qualitative sample*.* Three overarching themes describing the experience of the FSMP from both the patients’ and therapists’ perspectives emerged and included: barriers and facilitators to attending the FSMP; FSMP content, delivery and supporting documentation; and Trial processes.

**Table 5:** Patient participant characteristics

Patient participant characteristics		Number	Percentage
Gender	Male	2	15.38
	Female	11	84.62
Age	Mean (SD)	48.15 (16.69)	
	Range	23–83	
Trial Site	A	6	46.15
	B	7	53.85
Cohort	1	7	53.85
	2	6	46.15
Baseline Symptom Severity (FIQR)^a^	Mild	3	23.08
	Moderate	2	15.38
	Severe	5	38.46
	Extreme	3	23.08
Number of sessions attended	Mean (SD)	3.77 (2.09)	
	Range	0–6	

^a^Revised Fibromyalgia Impact Questionnaire (FIQR)

**Table 6:** Therapist participant characteristics with pseudonyms

Therapist	Trial site	Profession	Years qualified
Diane	A	Occupational Therapist	20
Georgia	A	Physiotherapist	7
Katie	B	Occupational Therapist	18
Mandy	B	Physiotherapist	10

#### Barriers and facilitators to attendance at the FSMP

The quantitative data highlighted that engagement with the intervention was low. Within the qualitative data the most cited barriers to attending the programme were travelling large distances and the cost of travel. Low attendance to the programme was most noticeable in the second cohort in Trial site B, where only two participants attended. The research team recruited participants up to 28 miles from the intervention site to secure sufficient participants to the second cohort in Trial site B. Travelling such large distances seemed to be unacceptable to participants.

‘[Trial site B] Which is a big old long stretch from here. Probably 45 minutes. And I don’t really think my £23 PIP is going to cover that taxi fare, do you?’ Linda, Trial site B

Other barriers that affected engagement with the intervention included FM flare, fatigue, unable to attend due to work and prior commitments. Factors that facilitated engagement with the intervention included the programme’s time; the delivery site was near their home; a supportive employer; and free parking.

#### FSMP content, delivery and supporting documentation

For those who engaged with the intervention, the course content was well received. The patient participants identified that the sessions on goal setting, pacing, the acceptance and grief cycle, relaxation and sleep were useful. Although the acceptance and grief cycle were perceived as helpful, some participants found discussing this overwhelming and others described feeling upset or angry.

‘Acceptance and grief. That just got me cross. It wasn't your fault and it wasn't anything to do with the course, it just made me realise how angry I still was about this bloody thing that has got in the way. And it did It affects your relationship and life and all sorts of things.’ Angela, Trial site A

To support the FSMP intervention, participants received a booklet, which provided an overview of the programme and further detail on each session. The therapists also used a similar booklet with additional information about leading the FSMP and both booklets were identified as valuable sources of information. Patient participants used the booklet as a legitimate source of information, facilitating communication about FM to family members and friends.

‘[The booklet] was brilliant. I was so impressed with that. There was so much information.’ Elizabeth, Trial site A

The participants who attended the FSMP found the therapists delivering the programme knowledgeable, friendly and helpful, and managed the group well. The therapists found adhering to the time allocated to each section in the programme challenging, as participants often asked additional questions. However, this depended on group size, previous patient participant experiences, and length of the session discussions.

Most participants reported they would attend the FSMP again and would recommend it to others. Some participants suggested the FSMP should be targeted to those recently diagnosed. Other suggestions were to include a session on employment rights, invite a friend or family member for support, include website links to recommended activities including Tai-Chi and implement the programme in more locations.

'The actual course, I would recommend it for anyone who has fibromyalgia plus for someone who is close to them who is having to deal with their fibromyalgia because it is very eye opening to them.’ Lisa, Trial site B

#### Trial processes

Overall, patient participants were positive about trial recruitment, screening and randomisation processes. All participants found the participant information leaflet and consent form acceptable and alternative formats could be considered for anyone with learning difficulties. The therapists, however, raised concerns regarding the eligibility criteria for some participants and queried their readiness for a group self-management programme.

'… myself and the OT felt they could have really done with some one-to-one or maybe they just weren't in the right place to be taking on self-management.’ Georgia, Physiotherapist

Patient participant experiences of completing the outcome measures were varied. Some participants did not experience any challenges, while others found them challenging to complete and were concerned about the length of time and concentration levels needed.

‘It was too much to do all in one, do you know what I mean. I don't know if I actually done it well because it's got lots of different points in it.’ Susan, Trial site B

All therapists delivering the FSMP found the training useful. However, therapists commented about the large amount of information provided within the two-day training and suggested that previous experience within pain management and intervention delivery contributed to their confidence and self-reported ability to deliver the sessions. Due to a minimal clinical experience of medication and diet, there was some anxiety about delivering these sessions. Further training on all areas for less experienced therapists was suggested.

## Discussion

Overall the trial was able to recruit patients with FM from primary care. However, as there were only four research-active GP sites in the county where Trial site B was located, there were challenges in recruiting the second cohort. In order to deliver the second FSMP cohort at Trial site B, participants were recruited from GP sites as far as 22 miles from the delivery site, which highlights the importance of sufficient research infrastructure to successfully identify eligible patients [57]. Another recruitment challenge in Trial site B was that many participants were not eligible to participate as they had previously attended the FSMP or a pain management programme. Overestimating the number of eligible participants is a common problem when recruiting to RCTs [58]. For a future RCT, it would be necessary to understand both the current provision of pain services and local research infrastructure when deciding upon trial intervention sites.

Attrition in trials is usually defined as high > 20% and low < 5% [59]. At 6 months, trial attrition in this feasibility study was 42%, and attrition was higher in the intervention compared to the control arm. Attrition to trials testing interventions that seek to change lifestyle often report attrition above 20% [60, 61]. A systematic literature review and meta-analysis showed there was, on average, slightly higher attrition in intervention arms of health behaviour change trials [62]. Additionally, the challenges of high attrition in FM treatment trials are noted by those testing drugs [63], group-based self-management [64] and exercise therapy [65]. one factor which may have affected attrition to the FALCON trial was that participants did not receive a clinical assessment before attending the FSMP in the community. This is different from the clinical service at the RUHB. Prior to attending the FSMP, patients participate in a 60-minute one-to-one therapy assessment where the therapist (OT or PT) discusses and agrees on treatment options, including attending the FSMP or one-to-one treatment. However, due to how community therapy services are currently delivered, it was not feasible to complete an hour clinical assessment before participants onward referral to the FSMP in the trial. The CI assessed eligibility to the trial, and the criteria broadly reflected clinical practice. Nevertheless, our findings suggest that it may be that the initial clinical assessment prepares FM patients for the FSMP and addresses the readiness of the patient to make changes. The therapist qualitative interviews also highlighted concerns that some patient-participants lacked preparedness to make behaviour changes. Previous research has also noted that further FM suitability screening based upon ASES-8 scores could help improve the patient retention of a community FM self-management programme [15]. Therefore, research investigating patients’ suitability of group-based FM self-management programmes and readiness to attend is required.

The qualitative results revealed that the main barriers to engagement with the intervention centred on travelling to attend, the cost of travel, and the exacerbation of FM symptoms. Factors that facilitated engagement with intervention included the time of the session, the programme locality, ease of parking, and a supportive employer. The cost of travel was a particular concern for those who received benefits. The NHS currently provides transport costs to attend NHS appointments and treatments to those on benefits. However, due to funding constraints, the feasibility study was unable to support patient-participants travel expenses. To replicate NHS practice, it is recommended that a future RCT should cover travel costs to the intervention for those participants who receive benefits. Travel concerns are commonly cited barriers to attending self-management interventions for musculoskeletal disease [66–69], and this will need to be considered for a full RCT. This may be more of a concern when delivering the intervention in rural sites compared to more urban areas as public transport is usually better for gaining access to city centres.

At the time of data collection, the FSMP was delivered face to face; however, in response to the COVID-19 pandemic, the clinical team at the RUHB adapted the FSMP programme to enable virtual delivery. Although there is little evidence to support or guide pain management programmes delivered virtually [70], eliminating the need to travel may increase intervention engagement. A recent systematic and meta-analysis into the self-management of chronic widespread pain, including FM, recommended further research into the mode of delivery, such as the internet, app or telephone-based [71]. Therefore, determining the clinical and cost-effectiveness of the virtual delivery of the FSMP is warranted.

### Proposed changes to the intervention

Data from the qualitative interviews suggest that the programme content, group delivery and the therapists delivering the intervention were acceptable. Those patient participants who did engage with the intervention reported improvements in managing their FM symptoms and would recommend the programme to others. One recommendation for change highlighted from the qualitative study was to include information about FM and employment. Research shows that FM affects a person’s ability to work [72] with an increased risk of unemployment and frequent need for additional support in the workplace [73, 74]. Therefore, the FSMP should be amended to include work-related information. The COVID-19 pandemic has changed how healthcare services are provided, with people becoming used to alternative delivery formats. We propose a future RCT test and evaluate how the FSMP is delivered.

### Proposed changes to the methodology

Eligibility criteria for the trial excluded all patients who had previously attended a pain management programme. The research team considered that those who previously participated at the FSMP in the recruitment site(s) or participated in a pain management programme would already have the skills to self-manage. Therefore, it is proposed that a full trial includes participants who attended a pain management programme > 12 months previously. Individuals with Rheumatoid Arthritis were excluded from the feasibility study, but those with other inflammatory rheumatic diseases such as, Ankylosing Spondylitis and Lupus were included. As FM is prevalent in several inflammatory rheumatic diseases and appears to affect disease severity [75], it is proposed that those with co-morbid FM and inflammatory rheumatic diseases, including Rheumatoid Arthritis, be included in a full RCT. We also propose that economic modelling should also reflect payment for travel to attend the FSMP for participants who receive benefits. One patient who could not communicate in English was excluded from the study, reflecting existing practice at the RUHB (patients unable to communicate in English are offered one-to-one self-management support with an NHS translator). However, we recognise that excluding those unable to communicate in English could exclude patients from ethnic backgrounds. A future RCT should consider recruiting these patients with translators and manuals adapted for other common ethnicities [76]. For the development of a future definitive trial, PPI members will be recruited to ensure that our research is meaningful to those living with FM. The research team will adhere to the INVOLVE framework of good practice for public involvement in research [77].

### Strengths

The study successfully recruited patients diagnosed with FM from primary care. As intended, the FSMP programme was delivered twice across two sites in SW England. This feasibility study has shown that it is possible to successfully train non-specialist therapists to deliver the FSMP in a community setting. Finally, the nested qualitative research provided an understanding of trial processes, including why some patients failed to engage with the FSMP and the acceptability of the FSMP from patient and therapist perspectives.

### Limitations

A limitation of the study was the high attrition rate (42%). Further to this, it was impossible to blind participants and treating therapists in this feasibility study. Blinding the participants and clinical staff will be challenging in further study. Blinding the data analyst would help to minimise the potential for bias in a full RCT [58].

Although the COVID-19 pandemic did not affect the delivery of this study, it may have delayed the return of the 6-month questionnaire data. Due to UK national lockdown, the study team received outcome data up to 4 months after the 6-month outcome data was initially sent. Additionally, although the medical record review was planned to be conducted by the RA in person, this was conducted via e-mail which impacted the timing of this study and may have impacted the data quality.

## Conclusion

It is feasible to recruit people living with FM from primary care to participate in an RCT testing clinical and cost-effectiveness of the FSMP delivered in a community setting. However, improvement in trial attrition and engagement with the intervention is needed. In addition, trial inclusion criteria should be refined to include those with inflammatory rheumatic diseases and those who have attended pain self-management programmes more than 12 months previously. An initial assessment by a therapist before attending the FSMP is also warranted to ensure patient readiness. Finally, it is suggested that a future trial incorporates an investigation of virtual delivery of the FSMP.

## Data Availability

The datasets generated during the current study are available in the University of the West of England Research Data Repository, http://researchdata.uwe.ac.uk/657.
